# Foams Set a New Pace for the Release of Diclofenac Sodium

**DOI:** 10.3390/pharmaceutics16020287

**Published:** 2024-02-18

**Authors:** Fanni Falusi, Szilvia Berkó, Mária Budai-Szűcs, Zoltán Veréb, Anita Kovács

**Affiliations:** 1 Institute of Pharmaceutical Technology and Regulatory Affairs, Faculty of Pharmacy, University of Szeged, 6 Eötvös St., 6720 Szeged, Hungary; falusi.fanni@szte.hu (F.F.); berko.szilvia@szte.hu (S.B.); budai-szucs.maria@szte.hu (M.B.-S.); 2 Regenerative Medicine and Cellular Pharmacology Laboratory, Department of Dermatology and Allergology, University of Szeged, 6720 Szeged, Hungary; vereb.zoltan@med.u-szeged.hu; 3 Centre of Excellence for Interdisciplinary Research, Development and Innovation, University of Szeged, 6720 Szeged, Hungary; 4 Hungarian Centre of Excellence for Molecular Medicine-USz Skin Research Group, University of Szeged, 6720 Szeged, Hungary

**Keywords:** dermal foam, diclofenac sodium, Raman mapping, in vitro permeation test, in vitro release test

## Abstract

Medicated foams have emerged as promising alternatives to traditional carrier systems in pharmaceutical research. Their rapid and convenient application allows for effective treatment of extensive or hirsute areas, as well as sensitive or inflamed skin surfaces. Foams possess excellent spreading capabilities on the skin, ensuring immediate drug absorption without the need for intense rubbing. Our research focuses on the comparison of physicochemical and biopharmaceutical properties of three drug delivery systems: foam, the foam bulk liquid, and a conventional hydrogel. During the development of the composition, widely used diclofenac sodium was employed. The safety of the formulae was confirmed through an in vitro cytotoxicity assay. Subsequently, the closed Franz diffusion cell was used to determine drug release and permeation in vitro. Ex vivo Raman spectroscopy was employed to investigate the presence of diclofenac sodium in various skin layers. The obtained results of the foam were compared to the bulk liquid and to a conventional hydrogel. In terms of drug release, the foam showed a rapid release, with 80% of diclofenac released within 30 min. In summary, the investigated foam holds promising potential as an alternative to traditional dermal carrier systems, offering faster drug release and permeation.

## 1. Introduction

Dermal drug delivery is a critical field of study in pharmaceutical research, with the goal of successfully administering therapeutic agents through the skin for localized or systemic effects.

A wide range of dermal preparations are available for use in product development. Within the traditional forms, solid, semi-solid, and liquid preparations are distinguished. Powders and patches are associated with the solid form applied dermally, while ointments, creams, gels, and pastes represent the semi-solid form. Solutions, emulsions, suspensions, and aerosols belong to the liquid form [1,2].

Only a few of the active ingredients are capable of achieving adequate transdermal penetration on their own since they need to possess suitable solubility and permeability [3]. To achieve a systemic effect, it is necessary to develop a formulation that is capable of crossing this protective barrier by temporarily disrupting the skin barrier before it quickly returns to its original structure. Among the methods for enhancing penetration, we distinguish between passive and active approaches. Passive methods involve reducing the barrier function of the stratum corneum through the use of chemical penetration enhancers [4,5], increasing hydration [6], and employing various nanostructured systems (NLC, liposome) [7].

In addition, an increasing number of new, innovative forms are being encountered in both the pharmaceutical and cosmetic industries [8]. In the field of dermatology, foams have gained attention [9,10], particularly in the treatment of sunburns, wounds, and ulcers. They are used in numerous areas, and the development of environmentally friendly designs has become paramount to reducing the environmental footprint [11]. This has led to the gradual replacement of propellant-containing systems with propellant-free systems. The therapeutic use of dermal foams is becoming increasingly appealing to the population because of its ease of application [12]. Foams are often utilized as a topical formulation, which allows for easier distribution and consistent covering of the affected region. Their appearance is aesthetic, non-greasy, and non-sticky, yet easily removable from the skin, thereby improving patient adherence. Foams also have good spreadability on the skin [13], ensuring the immediate absorption of the active ingredient, and eliminating the need for vigorous rubbing [14].

Foams have specific physical properties and a distinct structure that set them apart from other conventional drug delivery systems, such as hydrogels. Understanding the physical characteristics and structure of foams is critical for comprehending their benefits in dermal drug delivery. Foams are distinguished by their porous structure, which is formed during the foaming process [15]. These pores are bare spots or holes inside the foam matrix that contribute to its spongy appearance. The presence of pores in foams is essential for their drug delivery capabilities. The porous nature enables the incorporation and entrapment of drugs within the foam structure, allowing for sustained release upon [16,17,18] application to the skin. Additionally, this porous nature increases the accessible surface area for drug absorption, allowing for faster (immediate) drug diffusion. During formulation, it is essential to ensure the perfect dissolution of the active ingredient in the carrier excipient. Upon application, volatile components quickly evaporate from the foam applied to the skin, leading to supersaturation [19,20]. Consequently, a supersaturated layer forms on the epidermis in terms of the active ingredient, from which penetration initiates at a high speed due to the tremendous driving force within the system. If this process occurs rapidly, there is no opportunity for the active ingredient to crystallize since the rapid penetration causes a decrease in the concentration of the active ingredient in the foam layer. Furthermore, the linked network of foams allows for effective medication transportation across the epidermal layers, resulting in improved absorption and bioavailability [21].

Despite numerous advantages, formulating dermal foams presents significant challenges. When designing their compositions, it is crucial for the formulation to remain on the skin for a sufficient duration. It should quickly spread to meet user preferences and provide a pleasant skin sensation. In terms of shelf life, they are stored in sealed containers, minimizing microbiological contamination. However, despite the aforementioned advantages of foams, the number of available topical foam preparations in the market remains relatively low compared to traditional formulations such as creams and gels.

In our study, diclofenac sodium was used as an active pharmaceutical ingredient (API). Among the NSAIDs, diclofenac sodium is the only API approved by the FDA for topical use in the treatment of pain associated with osteoarthritis. Being an organic acid, diclofenac exhibits lipophilic characteristics, whereas its salts readily dissolve in water under neutral pH conditions. The mix of these two attributes enables diclofenac to effectively permeate cell membranes, encompassing the synovial lining of diarthrodial joints as well as the skin [22]. Furthermore, the occurrence of adverse effects is minimal compared to oral administration, especially those topical formulations that contain diclofenac [23,24]. Various concentrations of hydrogels, creams, and other products with this API are available on the market.

Foams and hydrogels are two significant rivals within the dermal field that have been widely researched. However, it is becoming increasingly clear that foams have triumphed, outperforming hydrogels in many ways and changing dermal drug delivery [21,25].

Foams possess excellent stability and a prolonged shelf life due to their ability to maintain structural integrity during storage and application. Unlike hydrogels, foams are less prone to leakage or drying out, ensuring a consistent and effective drug delivery performance over an extended period.

In summary, achieving transdermal permeation is challenging, requiring the development of formulations that can temporarily disrupt the skin barrier for systemic effects. Foams, gaining attention in dermatology, offer advantages such as an easy application, aesthetic appearance, and better patient adherence. Despite their numerous benefits, formulating dermal foams poses challenges, with considerations for duration on the skin, user preferences, and shelf life. In our study, diclofenac sodium served as an active substance, showcasing its effectiveness in topical formulations. In many ways, foams are superior to hydrogels in terms of stability and extended shelf life, making them a promising dosage form in dermal drug delivery research.

Our research focused on comparing the physicochemical and biopharmaceutical properties of three drug delivery systems: foam, foam bulk liquid (a polymer solution), and a conventional hydrogel. Addressing the limited studies on medicated foams, our goal was to develop comprehensive investigational methods covering aspects such as foam stability, viscosity, pH, in vitro drug release, and ex vivo skin permeation. This includes examining the potential differences in properties between the preparations, as well as investigating the impact of diclofenac sodium (DS) at a concentration of 1% on the foam system.

## 2. Materials and Methods

### 2.1. Materials

Diclofenac sodium (DS) and fluorescein sodium were handled by Sigma-Aldrich (Budapest, Hungary). Isopropanol (IPA) was obtained from Avantor (Radnor, PA, USA). Hydroxypropyl methylcellulose (HPMC) was provided by Colorcon (Budapest, Hungary). Polyethylene glycol 200 (PEG 200) was purchased from Merck KGaA (Darmstadt, Germany). Phenoxyethanol and Caprylyl Glycol were from Biesterfeld GmbH (Hamburg, Germany). Polyoxyl castor oil was kindly supplied by BASF SE Chemtrade GmbH (Ludwigshafen, Germany). Gattefossé (Saint-Priest Cedex, France) provided Caprylocaproyl Polyoxyl-8 glycerides and CP Kelco A Huber Company (Atlanta, GA, USA) provided xanthan gum. Deionized and purified water was used (Milli-Q system, Millipore, Milford, MA, USA) during the research. The cellulose acetate filter (Porafil membrane filter, cellulose acetate, pore diameter: 0.45 μm) was acquired from Macherey-Nagel GmbH & Co. KG (Düren, Germany). Additionally, 70% sodium laureth sulfate (SLES) was provided by Kao Chemicals Europe S.L. (Barcelona, Spain).

### 2.2. Methods

#### 2.2.1. Preparation of the Formulations

In terms of the examined formulations, both the foam and hydrogel contained the same non-ionic emulsifiers and preservatives at the same concentration. The difference lies in the type of the solvents and the type and the concentration of the polymer. In our preliminary research, we investigated several polymers. Among the foams, formulations containing xanthan gum exhibited the most stable and superior physicochemical properties [26], while for the hydrogels, those containing HPMC showed the best results.

The initial stage of hydrogel preparation involved the hydration of HPMC, which was carried out in purified water for a duration of 2 h. Simultaneously, a mixture of polyethylene glycol 200 and isopropanol was prepared. Following the swelling of the polymer, a predetermined quantity of DS was dissolved in the solvent mixture. The DS solution was then added incrementally to the hydrated polymer. The final homogenization of the formulation was carried out using a mechanical stirrer (Velp DLH Digital Overhead Stirrer, Italy). Ultimately, the uniform preparation was preserved.

The first stage of foam preparation was to prepare a polymer solution, with the polymers undergoing a 2 h swelling process in purified water. Subsequently, the preservative solution was blended with the emulsifiers. The final step involved incorporating the swelled polymer into the mixture of emulsifiers and preservative solution. Following the preparation and homogenization with a mechanical stirrer, the liquid was stored in a well-sealed container until the start of the examination. The process of preparing the bulk liquid and its composition in this study is identical to that of the foam.

The exact compositions are illustrated in Table 1.

#### 2.2.2. Citotoxicity Assay

The impact of the utilized components on cell toxicity was assessed through MTT assays following the manufacturer’s guidelines. Human-adipose-tissue-derived mesenchymal stem cells (AD-MSCs) were distributed into 96-well plates, with each well initially containing 5 × 10^3^ cells. These cells were then exposed to a solution containing the components, in the same concentrations as used in the formulations, for 24 h in triplicate. Absorbance was measured using the Synergy HTX multi-plate reader (Agilent/BioTek, Santa Clara, CA, USA) at 550 nm, with a reference wavelength set at 650 nm.

#### 2.2.3. Preformulation Studies of Foam Formula

In order to examine a foam formulation effectively, it is crucial to analyze the physicochemical properties of the foam formula. This analysis allows us to assess the impact of each component on the foam structure.

##### 2.2.3.1. Macroscopic Characterization of Foam Formula

The macroscopic characteristics of the foam formula were evaluated using the cylinder method [9]. After 5 min (min) of mechanical stirring of the bulk liquid, the foam was poured into a glass measuring cylinder, and the initial volume as well as the volume after 30 min of aging were measured. Macroscopic tests enable the determination of various parameters, including foam expansion (FE, %); foam volume stability (FVS, %); and foam liquid stability (FLS, %).

The parameters can be calculated using the following equations:

(1) $$\mathrm{FE}\left(\%\right)=\frac{V\left(\mathrm{foam}\right)-V\left(\mathrm{formulation}\right)}{V\left(\mathrm{formulation}\right)}\times 100\%$$

where V(formulation) represents the volume of the formulation [mL] required to generate V(foam) [mL]. A direct correlation can be observed between FE (foam expansion) and good foamability.

(2) $$\mathrm{FVS}\left(\%\right)=\frac{V\left(\mathrm{foam},\;30\;\min\right)}{V\left(\mathrm{foam}\right)}\times 100\%$$

where V(foam, 30 min) represents the volume of the foam after 30 min [mL].

(3) $$\mathrm{FLS}\left(\%\right)=\frac{V\left(\mathrm{liquid},\;30\;\min\right)}{V\left(\mathrm{foam}\right)}\times 100\%$$

where V(liquid, 30 min) is the drained volume after 30 min [mL].

##### 2.2.3.2. Microscopic Characterization of Foam Formula

The microscopic measurements were conducted using the Leica DM6 B Fully Automated Upright Microscope System (Leica Biosystems GmbH in Wetzlar, Germany). The structure of foams and the relative bubble sizes provide information on the differences between foam generation techniques and their stability. The images were captured at 50× magnification.

##### 2.2.3.3. Ex Vivo Permeation through the Skin Using Fluorescent Microscope

To model whether the formulation can permeate the stratum corneum, fluorescent microscopy was employed. During the investigation, the permeation capacity of the blank foam (without API) was examined. Experiments involving ex vivo skin permeation were carried out using excised human skin obtained from a Caucasian female patient who had undergone routine plastic surgery at the Department of Dermatology and Allergology, University of Szeged (Ethical Permission: BMEÜ/2339-3/2022/EKU). Following the plastic surgery, the skin surface underwent a gentle cleansing process using cotton swabs and was subsequently stored at a temperature of −20 °C for a maximum period of 6 months before being used.

Fluorescein sodium water-soluble dye was used to visualize the permeation of the foam system. At room temperature, full-thickness subcutaneous fat-free human abdominal skin was used in the experiment. The skin samples were defrosted and kept on filter papers soaked in a phosphate-buffered solution to preserve their hydration. To ensure the permeation of formulations, 0.2 g of each formulation was applied to the skin surface, and observation times of 10 and 30 min were employed. Following the treatment, any excess preparation remaining on the skin was carefully wiped off. Subsequently, a section of the treated skin was frozen and sliced using a Leica CM1950 Cryostat (Leica Biosystems GmbH, Wetzlar, Germany). Cross-sections with a thickness of 10 μm were placed on slides and examined using a light microscope (LEICA DM6 B, Leica Microsystems GmbH, Wetzlar, Germany) at room temperature. A red fluorescence filter (580–660 nm) was utilized to prevent interference from skin autofluorescence during the analysis. The examination was conducted at a magnification of 200× [4].

Images of the untreated skin were captured as a negative control, while skin pretreated with a solution containing sodium laureth sulfate (SLES) was used as a positive control. Images of the treatments were taken and visually compared to the control groups. ImageJ1 software was employed to assess the color intensity of the images, representing the distribution of color intensity within each image. The increase in intensity is indicated as relative intensity (RI), signifying how many times the increase in intensity compares to the negative control (untreated skin) [27].

#### 2.2.4. Comparison of Physicochemical Properties of Foam Formula, Bulk Liquid, and Hydrogel

##### 2.2.4.1. Rheological Measurements

The viscosity of the bulk liquid, foam, and hydrogel was examined using an Anton Paar Physica MCR302 Rheometer (Anton Paar, Graz, Austria) at a temperature of 25 °C. A cone-plate-type measuring device was applied with a diameter of 50 mm, and the gap height in the middle of the cone was 0.045 mm. The RheoCompass™ software v.1.25 (Anton Paar, Graz, Austria) of the instrument was utilized to calculate the viscosity of the preparations at a shear rate of 50 1/s through interpolation. The process involved conducting three measurements in parallel. Flow curves of the investigated formulations were plotted from a 0.1 to 100 1/s shear rate.

##### 2.2.4.2. Investigation of pH

Each 5 g sample was placed in a beaker, and the pH was measured using a Testo 206 pH meter (Testo SE & Co. KGaA, Lenzkirch, Germany), at room temperature. Three measurements were performed in parallel. The pH values were evaluated to assess the basicity/acidity of the preparations since the normal pH of the skin varies from 4 to 6 [28,29,30].

#### 2.2.5. Comparison of Biopharmaceutical Properties of Foam Formula, Bulk Liquid, and Hydrogel

##### 2.2.5.1. In Vitro Drug Release and Permeation Tests (IVRT and IVPT) Using Franz Diffusion Cell System

The drug release through the synthetic membrane from the bulk liquid, foam, and hydrogel, as well as its permeation through the human heat-separated epidermis, were modeled using the Vertical Franz diffusion cell (Hanson Microette TM Topical & Transdermal Diffusion Cell System, Hanson Research Corporation, Chatsworth, CA, USA). The excised human skin, just like in the case of the fluorescent microscope method, was obtained through plastic surgery.

For in vitro release tests, as a donor phase, 0.3 g of the sample (in the case of the hydrogel and bulk liquid) was applied onto a synthetic membrane filter. In the case of foam (due to its large volume), 0.085 g was placed onto the membrane (Porafil cellulose acetate membrane with a pore diameter of 0.45 μm, Macherey-Nagel GmbH & Co. KG, Düren, Germany). In contrast, for the in vitro permeation test, a heat-separated human epidermis [31,32] was employed as the membrane. Both the drug release and permeation tests lasted 6 h and the sampling dates were 10, 20, and 30 min, and 1, 2, 4, and 6 h. The amount of the active pharmaceutical ingredient released from the formulation and transported through the skin was determined by using UHPLC (Shimadzu Nexera ×2, Shimadzu, Kyoto, Japan, ultra high-performance liquid chromatography system).

The UHPLC was equipped with a Phenomenex Kinetex XB-C18 (50 × 2.1 mm, 2.6 µm) column, which was used as a stationary phase. Separation was achieved through isocratic elution, employing a 36:64 mixture of a 0.136 g/L KH_2_PO_4_ solution and methanol as the eluent. The separation procedure occurred at 40 °C with a flow rate of 0.5 mL/min, spanning a 3 min analysis time. The retention time for DS was noted at 1.5 min. A sample volume of 3 µL was injected for the analysis. Detection was carried out using a diode array UV-VIS detector at a wavelength of 247 nm [33].

To study the release mechanism of the investigated formulations, we applied the Korsmeyer–Peppas model by fitting it to the release curve until reaching the plateau phase, providing information about the mechanism of drug release [34].

The calculation of in vitro permeation was based on the cumulative amount of DS that permeated through the epidermis, considering the diffusion area. These findings were graphed over time, and the steady-state flux (J) was calculated from the slope of the permeation curve, quantified in terms of μg cm^−2^ h^−1^. For this analysis, the incubation period ranged from 1 to 6 h, during which the flux data for DS were determined.

##### 2.2.5.2. Investigation of Ex Vivo Drug Permeation Using Raman Spectroscopy

Raman spectroscopy is an emerging spectroscopic approach grounded in identifying the characteristic vibrational energy states of a molecule when exposed to laser irradiation. It offers insights into the molecular arrangement of tissue constituents devoid of the necessity for fluorescent markers or chemical dyes [35,36]. The confocal Raman microscopy can be employed to investigate topical formulations, for determining both permeation and permeation depth. In our research, Raman microscopy was utilized to capture images depicting the spatial distribution of DS within ex vivo human skin.

The preparation and sectioning of the skin were conducted in the same manner as for fluorescence microscopy examination, with the only difference being the incubation time of 3 h. Subsequently, the cross-sectional skin samples, with a thickness of approximately 15 micrometers, were positioned onto slides coated with aluminum. Raman spectroscopic assessments were conducted using a Thermo Fisher DXR Dispersive Raman Spectrometer (Thermo Fisher Scientific Inc., Waltham, MA, USA) fitted with a CCD camera and a diode laser. A laser light emitting at a wavelength of 780 nm was employed, reaching a peak power of 24 mW. This wavelength is optimal for studying biological specimens as it provides enough energy for the vibrations of protein constituents within the skin. Using this specific laser source reduced the impact of fluorescence. For the measurements, a microscopic lens with a magnification of 50× was employed, and the pinhole aperture had a diameter of 25 μm [37]. While conducting the mapping procedure, an area of 100 × 500 μm on the skin was visualized, using both vertical and horizontal step sizes of 50 μm. Throughout the measurement, the map of the untreated skin was used as a control.

The spectra of DS in the bulk liquid and hydrogel were employed as a basis for comparing treated and untreated skin samples. To capture the spectra of DS, a 780 nm laser source was utilized. A total of 33 scans were recorded for each spectrum with an exposure time of 6 s. The Raman microscope featured 10× optical magnification with a 25 μm slit aperture.

The data collection and analysis were carried out using the Dispersive Raman software package OMNICTM 8.2 (ThermoFisher Scientific Inc., Waltham, MA, USA).

#### 2.2.6. Statistical Analysis

The results of the in vitro drug release tests underwent statistical evaluation using the two-way ANOVA analysis of variance test (Bonferroni’s multiple comparison) with Prism 5.0 for Windows 10 software (GraphPad Software Inc., La Jolla, CA, USA). The data represent the mean values derived from six experiments, along with the standard deviations, and significant differences from the foam formulation were observed at the levels of * *p* ≤ 0.05 and *** p ≤ 0.001.

## 3. Results

### 3.1. Cytotoxicity Assay

**Figure 1:**
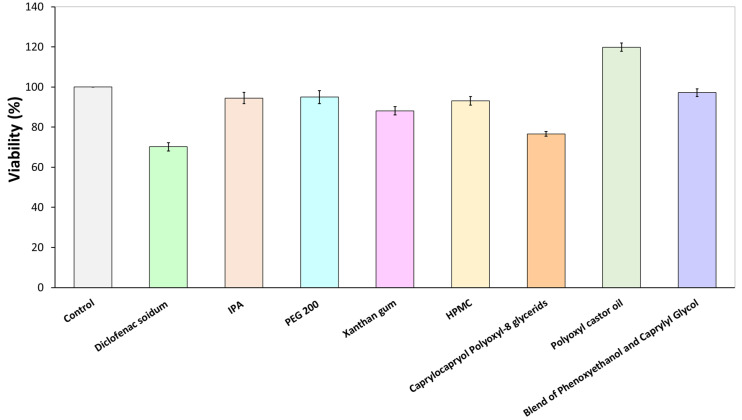
Effect of components on the viability of AD-MSCs.

The MTT test was conducted for the components of all three examined preparations (Figure 1). The findings indicated that the applied components enhanced cell viability, although diclofenac sodium had a minor reducing effect, as determined by the MTT assay. All examined components exhibited a viability of over 70%, indicating that, in accordance with the ISO 10993-5 standard [38], these substances are not cytotoxic to mesenchymal cells. The control group demonstrated 100% viability (measured in triplicate, N = 3).

### 3.2. Preformulation Studies of Foam Formula

A preformulation study was carried out to ensure that our foam formula meets the physicochemical and biopharmaceutical properties required for dermal application.

#### 3.2.1. Macroscopic Characterization of Foam Formula

In order to determine the effect of the polymer and the active pharmaceutical ingredient (API) on foam expansion, foam volume, and foam liquid stability, a comparison was made between a polymer-and-active-ingredient-free formulation, a xanthan-gum-containing, active-ingredient-free formulation, and the foam system containing DS and xanthan gum together (Table 2).

The API and the polymer content had a negative effect on foam expansion, possibly due to the initial increase in the viscosity of the bulk liquid. However, each foam formulation met the criterion for well-foaming preparations, exhibiting over 100% foam expansion [26].

Regarding foam volume stability, macroscopic observations clearly supported that the polymer-and-API-free systems collapsed quickly, while the xanthan-gum-containing system maintained its foam volume even after 30 min. The foam system containing DS, which also included xanthan gum as a polymer, also preserved its original volume at nearly 100%.

The FLS value also indicates macroscopic foam stability and a lower FLS value refers to better stability. In this case as well, it was observed that the polymer-free foam had lower stability, compared to the other two formulations.

#### 3.2.2. Microscopic Characterization of Foam Formula

The structure of foams can be analyzed directly after their formation using a light microscope. The examination of the microscopic structure of foams can be conducted most conveniently with a fully automated microscopic system. Microscopic images provide the opportunity to study the connections between bubbles and liquid films, known as lamellae, that enclose the bubbles. Additionally, the changes in bubble size and number over time can be observed.

**Figure 2:**
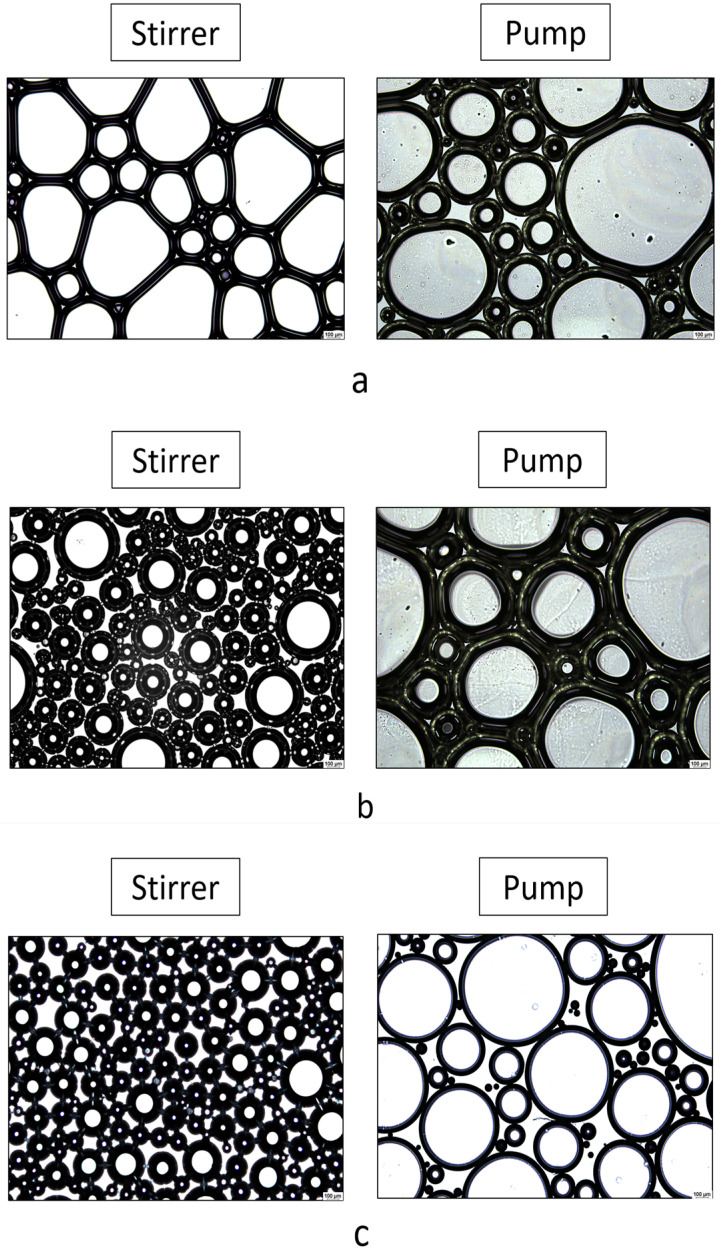
Microscopic images of the structure of foams produced by the pump and mechanical stirrer: (**a**) polymer- and DS-free foams; (**b**) xanthan-gum-containing, DS-free foam; (**c**) xanthan-gum- and DS-containing foam. The images were captured at 50× magnification.

The foams were produced using two different foam formation techniques, which were compared during the investigation (Figure 2). Mechanical stirring provides a more realistic representation of foam formation, and the duration of foam formation can be better tracked, while the foams produced with a propellant-free pump simulate real application conditions.

The bubbles produced with a propeller stirrer are smaller and more uniform in size in the case of xanthan-gum- and diclofenac-containing formulations. The smaller bubbles contribute to the formation of a coherent foam structure, making these systems more stable than the polymer- and API-free formulation. The results, therefore, correlate with the results of macroscopic foam stability. For the formulations produced with the pump, the film thickness was greater in the case of xanthan-gum-containing compositions, and the amount of liquid bound by the polymer in the boundary layer could be more significant.

#### 3.2.3. Ex Vivo Permeation through Fluorescent Microscope

**Figure 3:**
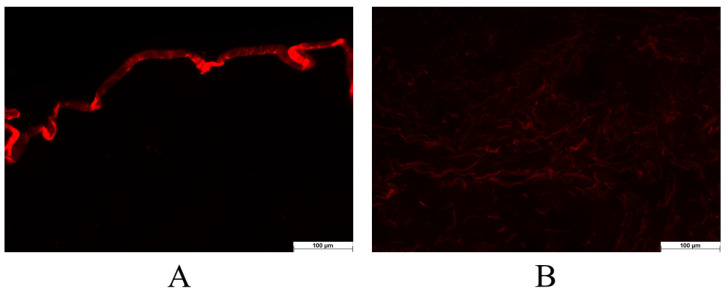
The untreated skin was used as a negative control. The stratum corneum with the upper skin layers (**A**) and the lower skin layers (**B**). The examination was conducted at a magnification of 200×.

The foam was compared to the negative and positive controls throughout the investigation. The negative control involved assessing the appearance of untreated skin under a fluorescent microscope. The microscopical images revealed that the stratum corneum exhibited a high fluorescence intensity (Figure 3A). This characteristic of untreated skin has already been documented in previous literature, which indicates the physiological appearance of the structure of the stratum corneum [39]. To determine the permeation of the formulation marked with the fluorescent dye, lower epidermal and dermal layers need to be examined since these layers only appear with low intensity under the fluorescent filter, and the autofluorescence of the skin does not interfere with the evaluation (Figure 3B).

In the case of the positive control, the skin was pretreated with an SLES solution, which facilitated permeation. Alkyl sulfates have the capacity to disrupt the barrier structure and allow a fluorescein dye solution to pass through the stratum corneum. Based on these evaluations, the increase in intensity (relative intensity) was assessed compared to the untreated skin (negative control).

**Figure 4:**
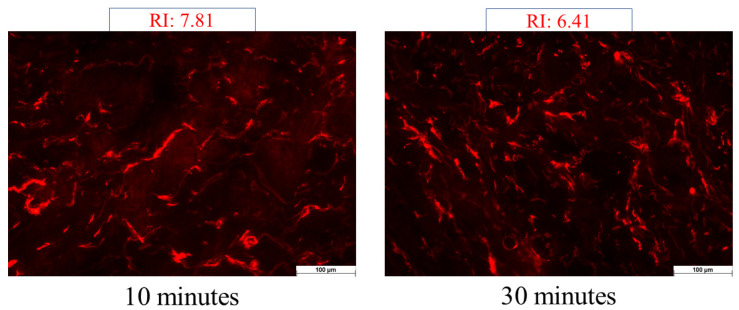
Images of the positive control: Relative Intensity Values at 10 and 30 min. The examination was conducted at a magnification of 200×.

The findings indicated (seen in Figure 4) that the light intensity of the skin significantly increased following the SLES pretreatment. SLES reduced the protective function of the stratum corneum, allowing the fluorescent dye solution to reach deeper layers of the skin. After 10 min of treatment, the intensity increased by 7.51 times, while after 30 min of treatment, it only increased by 6.41 times compared to the negative control.

**Figure 5:**
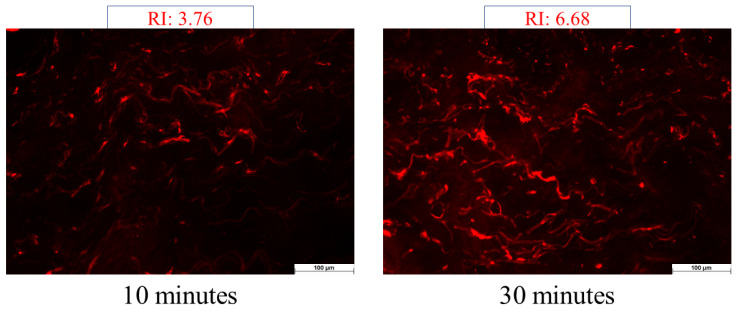
Images of the skin treated with the foam formulation: Relative Intensity Values at 10 and 30 min. The examination was conducted at a magnification of 200×.

In the case of the foam, as the observation time increased, there was a noticeable increase in fluorescence intensity. After 10 min, there was a 3.76-fold increase in permeation, but after 30 min, this value increased by 6.68-fold (Figure 5). In the case of foam preparation, the intensity of permeation was low at 10 min, but after 30 min, it reached almost the same relative intensity compared to the positive control. Without irritation, deeper permeation similar to the positive control can be achieved with the foam formulation.

### 3.3. Comparison of Physicochemical Properties of Foam Formula, Bulk Liquid, and Hydrogel

#### 3.3.1. Rheological Measurements

The consistency of the systems was investigated with rheological measurements.

The viscosities of the bulk liquid and the liquid film, remaining after the breakdown of the foam, were compared to each other and the reference hydrogel formulation (Table 3).

The foam was formed from the bulk liquid (initial polymer solution), during which the propellant-free foam pump mixed it with the ambient air. After a certain period of time, as the foam decayed, it transformed into a liquid film through the effects of binding forces and interactions between chains, causing the polymer chains to form a more orderly network or structure than what exists among the polymers in the initial liquid. This ordered structure can result in reduced volume filling and density, increasing the viscosity of the liquid film. The viscosity of the formulated hydrogel was much higher, found to be typical of semi-solid formulations.

According to the rheological results (Figure S1), all three systems exhibit the characteristic shear-thinning behavior typical of polymer solutions, where viscosity decreases under the influence of shear. Clearly, in the case of the hydrogel, the viscosity value is high due to the higher polymer content. The viscosity of the remaining liquid film was greater than that of the initial liquid. The increase in viscosity may be due to the ordered structure of the polymer liquid film, making it less liquid and more resistant to deformation. This could potentially cause a slight delay in skin permeation due to the more orderly network formed between polymer chains.

#### 3.3.2. Investigation of pH

Testing and adjusting the pH of dermal formulations can be key to ensuring the efficacy of the formulation and the barrier function of the skin. The pH values of the bulk liquid/foam and the hydrogel were in the range of 7–8, revealing the suitability of them for topical application, as it is reported in Table 4. In addition, the surface of the stratum corneum is slightly acidic, although it tends toward more neutral values (pH 7–7.4) in the vital layers [40]. Moreover, in the short term, a higher pH value may be tolerated. Human skin has a certain degree of buffer capacity and may tolerate a slight pH change in the skin.

### 3.4. Comparison of Biopharmaceutical Properties of Foam Formula, Bulk Liquid, and Hydrogel

#### 3.4.1. In Vitro Drug Release and Permeation Tests (IVRT and IVPT) Using Franz Diffusion Cell System

**Figure 6:**
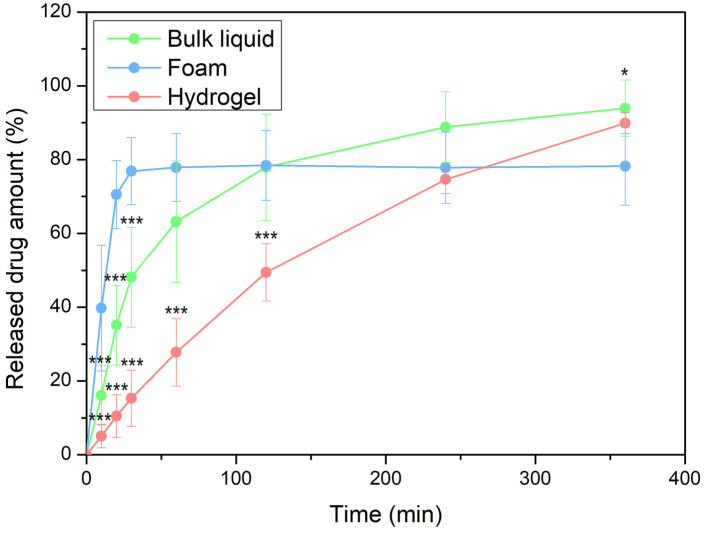
The release profile of DS in different dosage forms (*** *p* ≤ 0.001 vs. foam, * *p* ≤ 0.05 vs. foam).

The release of the active substance may depend on a number of factors that can influence how much and at what rate it is released from a given formulation into the surrounding medium or onto the treated surface. The drug release curves of the three forms are shown in Figure 6.

The results suggested that most of the DS was released from the foam within a relatively brief duration. Around 80% of the active ingredient was released in just 30 min when using the foam, whereas it took approximately 5 h for the hydrogel to achieve the same outcome. This quantity of DS was released from the bulk liquid after approximately 2 h.

The results could be due to the porous structure of the foam; it consists of many pores, channels, or air bubbles in which the active substances are more easily dispersed. This porous structure allows the active ingredients to move and reach their target site more quickly. The kinetics of release exhibited a resemblance between the bulk liquid and the hydrogel; however, DS demonstrated a slightly more rapid diffusion from the bulk liquid. On the one hand, polymer solutions (bulk liquid) generally have a lower viscosity than hydrogels. The lower viscosity allows the active molecules to diffuse more easily from the polymer solution into the surrounding medium or onto the skin. Conversely, hydrogels have a higher viscosity, which may limit the diffusion and release of the active substance.

On the other hand, the release may be affected by the polymer network and structure. Hydrogels typically possess a more organized, interconnected structure, which may result in a slower release as the drug has more difficulty diffusing through the polymer network.

In the investigation, the release of the drug is comprehensively studied and explained through the application of the Korsmeyer–Peppas model. This model allows for a detailed analysis and understanding of the drug release kinetics, providing valuable insights into the release mechanism (Table 5).

In the case of all systems, the observed *n* values are between 0.5 and 1.0, indicating that the mechanism of drug transport involves both diffusion and relaxation (erosion). Based on the k value, the fastest release rate was observed in the case of foam formulation [34].

The permeation rate values (Table 6) suggested that, in the case of foam, quick drug release resulted in rapid drug permeation. The foam can come into contact with the skin over a large surface area and the active ingredient can be absorbed quickly. This facilitates the swift delivery of active ingredients to the skin, expediting their effectiveness. The liquid film that emerges after the foam decaying may become supersaturated, resulting in accelerated permeation compared to hydrogel and bulk liquid.

#### 3.4.2. Investigation of Ex Vivo Drug Permeation Using Raman Spectroscopy

**Figure 7:**
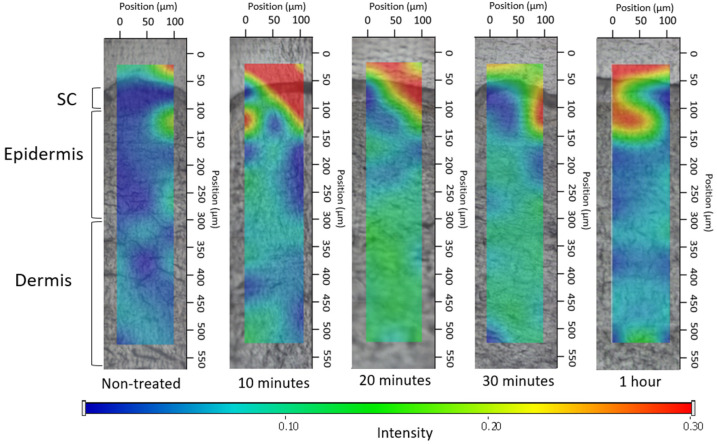
Kinetics of ex vivo drug permeation of bulk liquid.

**Figure 8:**
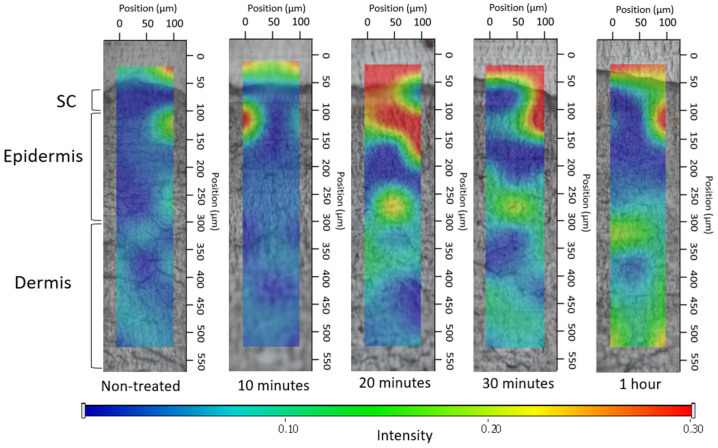
Kinetics of ex vivo drug permeation of foam.

The correlation maps depicted the distribution of DS, employing suitable spectra for accurate fitting with the spectra of the treated skin. Figure 6, Figure 7 and Figure 8 exhibit the qualitative distribution of DS within human skin samples following the application of foam, bulk liquid, and hydrogel. Our objective was to ascertain whether the permeation of DS remains confined to the stratum corneum or if it can permeate into the epidermis or dermis. On the maps, the warmer color indicates a higher presence of DS.

For the bulk liquid, DS became detectable in the deeper skin layers within 10 min and exhibited a more pronounced presence in these layers by 30 min (Figure 7). In contrast, in the case of skin sections treated with foam, the DS was concentrated in the upper layers of the epidermis after 10 min, and the presence of the active substance was detected in deeper layers as time progressed (Figure 8). Due to the gradual decay of the foam, liquid between the bubbles began to leak onto the skin after 10 min, forming a supersaturated liquid layer. Consequently, the intense presence of DS in the foam became more prominent, with higher concentrations of the active substance observed between 20 and 30 min.

**Figure 9:**
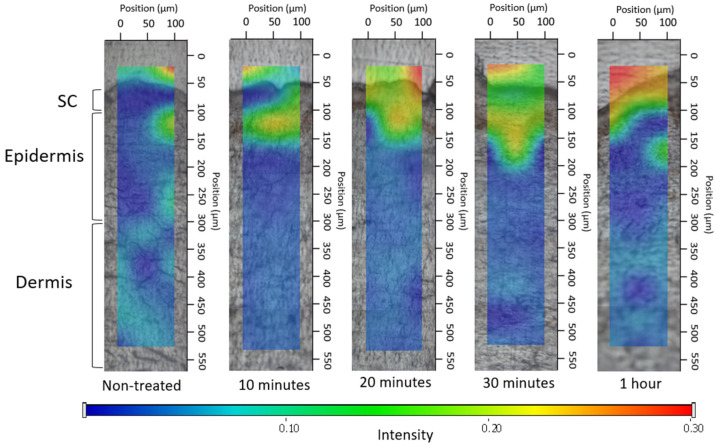
Kinetics of ex vivo drug permeation of hydrogel.

Regarding the hydrogel (Figure 9), the observation indicated that DS managed to permeate solely into the uppermost epidermal layer throughout the study duration. After 1 h, higher concentrations were achieved in the stratum corneum.

To conclude, the Raman maps highlighted the impact of viscosity on permeation. The composition of the foam and bulk liquid is the same; however, in the case of the foam formula, there is a higher permeation of the active ingredient after 20 min, which is visible as an intense red color on the Raman map. When compared to the hydrogel, possessing the greatest viscosity, it hindered the permeation of DS; therefore, it did not permeate the deeper layers of the skin even after 1 h. This higher viscosity prevents the active ingredient from permeating into the deeper layers of the skin even after 1 h. Meanwhile, the bulk liquid, with the lowest viscosity, exhibited swift and intense permeation into the deeper layers. The formation of a supersaturated liquid film during foam aging was evident after 20 min and its effects were still detectable after 1 h. The supersaturated liquid film formed during the aging of the foam could be formed after 20 min and its effect was still detectable after 1 h.

## 4. Discussion

In this work, we compared the physicochemical and biopharmaceutical properties of foams with those of traditional hydrogel and polymer solutions.

According to the cytotoxicity assessment, the components in the formulations did not exhibit any cytotoxic impact on mesenchymal cells at the concentrations used. Therefore, we found these components to be suitable for the formulation of dermally applied preparations.

In terms of preformulation studies of foams, the presence of DS reduced foam expansion based on macroscopic observations but did not negatively impact foam stability, as confirmed by microscopic results, since it had no adverse effects on the foam structure. The pH values of both the bulk liquid/foam and the hydrogel ranged from 7 to 8, making them suitable for topical application.

Biopharmaceutical examinations revealed that the foam, as a drug delivery system, can achieve rapid drug release and deeper skin permeation compared to the hydrogel. Approximately 80% of the active ingredient was released in just 30 min using the foam, while it took approximately 5 h for the hydrogel to achieve the same outcome.

The drug release from the formulations was studied and explained using the Korsmeyer–Peppas model. The fastest release rate was observed in the case of foam formulation, which correlates with the result of drug permeation.

Results from Raman skin permeation studies demonstrated that within just 10 min, the foam concentrated in the upper layers of the epidermis and gradually permeated even deeper layers over time. The supersaturated liquid film formed during the aging of the foam could be observed after 20 min, and its effect was still detectable after 1 h. The Raman mapping results exhibited a strong correlation with the fluorescent microscopic examination, as the foam formulation maintained high light intensity even after 10 min, providing additional evidence for the system’s rapid permeation. In comparison, the hydrogel, with the greatest viscosity, hindered the permeation of DS. Therefore, it did not permeate the deeper layers of the skin even after 1 h.

The applied test methods were suitable for the complex investigation of the foam formula, including the physicochemical and biopharmaceutical properties, as well as for the detection of the potential differences between the preparations. Furthermore, it can be concluded that diclofenac sodium (DS) at a concentration of 1% did not negatively affect the stability of the foam.

## 5. Conclusions

Overall, foaming systems have great potential through rapid drug release and deeper skin permeation, not only for the pharmaceutical industry but also for the cosmetic industry. Among our future plans is the assessment of the developed formulations’ effects on keratinocytes and mesenchymal stem cells in vitro. Additionally, we aim to conduct in vivo studies using animal models.

## Figures and Tables

**Table 1:** Composition of the formulated foam, foam bulk liquid, and hydrogel (‘+’ indicates that the formulation contains the excipient, ‘−’ indicates that the formulation does not contain the excipient).

	Hydrogel	Foam/Foam Bulk Liquid
Diclofenac sodium (g)	1	1
PEG 200	+	−
IPA	+	−
HPMC	+	−
Xanthan gum	−	+
Caprylocaproyl Polyoxyl-8 glycerides	+	+
Polyoxyl castor oil	+	+
Blend of Phenoxyethanol and Caprylyl Glycol	+	+
Purified water	up to 100 g	up to 100 g

**Table 2:** Result of the macroscopic examination.

	Polymer-and-API-Free Foam	Xanthan-Gum-Containing Foam	Xanthan-Gum-and-DS-Containing Foam
Foam expansion (FE, %)	172 ±15.8	134 ± 1.9	120 ± 0.7
Foam volume stability (FVS, %)	14 ± 1.8	100 ±0.0	98 ± 0.0
Foam liquid stability (FLS, %)	36 ± 2.0	0 ± 0.0	1.5 ± 0.2

**Table 3:** The viscosity of the formulated preparations at 50 1/s shear rate.

	Viscosity (mPas)
Bulk liquid	46.99 ± 0.80
Liquid film (after the foam decay)	58.68 ± 1.07
Hydrogel	378.78 ± 4.39

**Table 4:** The pH values of the formulated preparations.

	pH
Bulk liquid/Foam	7.86 ± 0.11
Hydrogel	7.29 ± 0.37

**Table 5:** Korsmeyer–Peppas model for the mechanism of drug release kinetics of the investigated formulations.

Formulation	R^2^	n	k
Bulk liquid	0.9094	0.6105	4.9352
Foam	0.9372	0.6243	9.8115
Hydrogel	0.9880	0.8033	0.9284

**Table 6:** The flux values of DS through the human epidermis.

	J (µg/cm^2^/h)	R^2^
Bulk liquid	0.866	0.9811
Foam	5.838	0.9921
Hydrogel	1.65	0.9173

## Data Availability

The data presented in this study are available on request from the corresponding author.

## Supplementary Materials

The following supporting information can be downloaded at: https://www.mdpi.com/article/10.3390/pharmaceutics16020287/s1, Figure S1: Flow curves of the investigated systems.
