# Supplementary material for: Foams Set a New Pace for the Release of Diclofenac Sodium
Source: Pharmaceutics. 2024 Feb 18;16(2):287. doi: 10.3390/pharmaceutics16020287 (PMC10892945; doi:10.3390/pharmaceutics16020287)
Supplement: Supplementary file 1 [file pharmaceutics-16-00287-s001.zip › pharmaceutics-2855251-supplementary.pdf]

Supplementary Material

Foams set a new pace for the release of diclofenac sodium

Fanni Falusi[1], Szilvia Berkó[1], Mária Budai-Szűcs[1], Zoltán Veréb[2,3,4], Anita Kovács[1*]

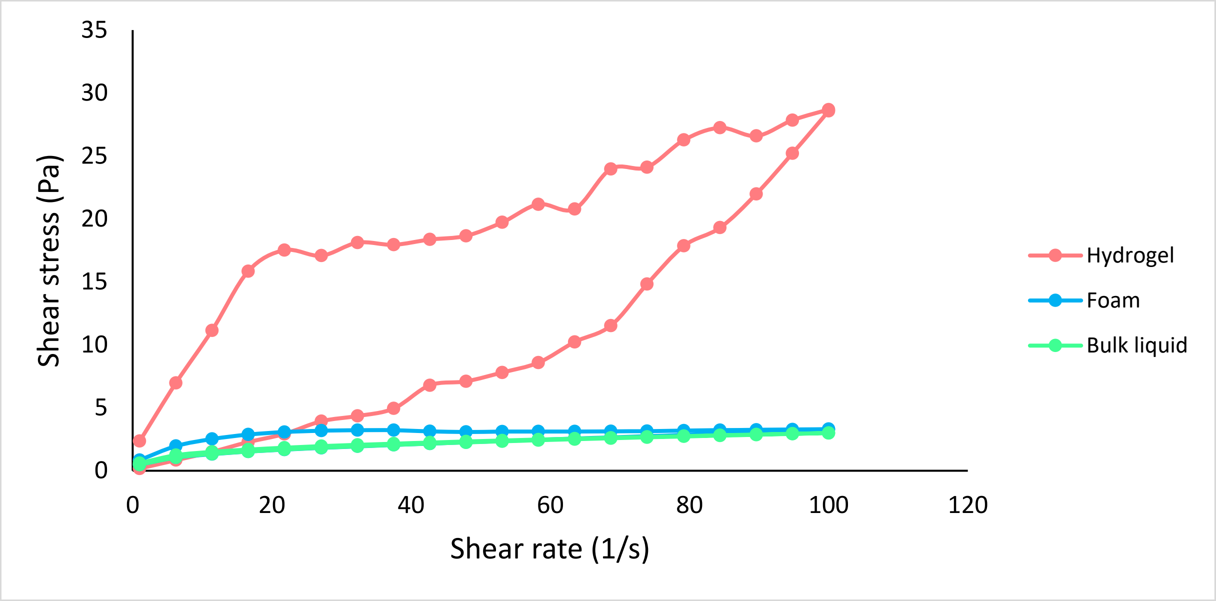

Figure S1. Flow curves of the investigated systems.
